# Psychological ailments and their treatment protocols: a case study of Swati traditional healers in Mpumalanga Province, South Africa

**DOI:** 10.4314/ahs.v21i2.50

**Published:** 2021-06

**Authors:** Anastasia Ngobe, Sebua Semenya, Tholene Sodi

**Affiliations:** 1 University of Limpopo, Research Administration and Development; 2 University of Limpopo, Psychology

**Keywords:** Mental illness, Mpumalanga, Swati, traditional healers, treatment methods, psychological ailments

## Abstract

**Background:**

Evidence suggests that South African traditional healers (THs) treat various mental complaints. However, there is little literature on Swati THs' accounts on this subject. The current study therefore, sought to address this gap.

**Methods:**

Data was gathered using qualitative research methods, namely semi-structured interviews with 10 purposely sampled Swati THs practicing in the Kanyamazane peri-urban township (Mpumalanga Province, South Africa). Data was thematically analysed.

**Results:**

Results showed that THs treat seven psychological aliments, viz. adjustment disorders, depression, mental illness due to ancestral calling, mental illness due to bewitchment, mental illness due to breaking of taboos, psychotic disturbance and substance induced mental illness. Generally, an integrated treatment protocol was utilised by THs to treat and manage these disorders. Most of these procedures are acceptable from either folkloric or scientific viewpoint, and have demonstrated certain level of efficacy in treating mental illness.

**Conclusion:**

Taken together, the evidence presented indicates that Swati THs use different traditional methods to manage various mental complaints. In doing so, they carry a large share of the community caseload for mental health, whilst admitting patients in their homes for extended periods of time, and also referring some (patients) for additional care within the Western health sector.

## Introduction

A mental illness is a health problem that significantly affects how a person feels, thinks and behaves, thus diminishing one's capacity for coping with ordinary demands of life^1^. The most common mental disorders encompass depression, generalized anxiety disorder, panic disorder, phobas, social anxiety disorder, obsessive-compulsive disorder, and post-traumatic stress disorder^2^. Globally, approximately 450 million people suffer from one or more of these disorders, and almost 1 million people commit suicide every year due to these disorders^3^. Nearly 50 percent of Americans have been mentally ill at some point in their lives, and more than a quarter have suffered from mental illness in the past twelve months^4^. The national prevalence of mental disorders in Brazil ranges from three to 52.2%^5^. In Japan, Nishio et al.^6^ found that roughly 61% of homeless people are diagnosed with mental disorders.

Mental disorders are also highly prevalent in Africa. For instance, Jenkins et al.^7^ reported the prevalence of 10.8% amongst the population of Kenya, largely comprising mixed anxiety depression, panic disorder, generalized anxiety disorder and depressive episodes. In Uganda, mental illness such as depression, anxiety and bipolar disorder is 9.3%, 8.5%, and 4.9% respectively^8^. Out of 80% of Congolese patients diagnosed with mental illness, the majority have schizophrenia, anxiety and mood disorders^9^. A national survey conducted by Herma et al.^10^ across the nine provinces of South Africa found that at least one in five South Africans is adversely affected by mental disorders, with common ones being major depression (9,8%), and agoraphobia (9,8%). Other epidemiological studies restricted to certain provinces of South Africa also indicated that mental disorders are rampant^11^,^12^.

Generally, mental health problems are associated with multi-faceted factors including social changes, work stress, discriminations, social exclusions, poor lifestyles, risk of violence and physical illnesses^13^. Therefore, elimination of some of these factors will contribute significantly towards the management of mental disorders. Mental illnesses can be effectively treated and managed using pharmacological and psychosocial treatments. Pharmacological treatments include Amitriptyline, Carbamazepine, Chlorpromazine, Clomipramine, Diazepam, Fluoxetine, Fluphenazine, Haloperidol and Lithium carbonate^14^. Psychosocial treatment of mental illness entails crisis management, psychotherapy such as cognitive-behavioural, supportive, and family therapy, and usually outpatient treatment where appropriate^15^. In Africa, traditional healers (THs) play a vital role in the health care of the majority of the people^16^. Therefore, it is not surprising that approximately half of African population seeking formal health care for mental disorders consult THs as their first line of care^17^,^18^,^19^,^20^,^21^. Traditional healing for the treatment of mental illness is preferred for a number of reasons including its accessibility, affordability and its philosophy of seeing the person holistically rather than through a Cartesian divide^22^.

According to Chipfakacha^23^ all cultures have disease theory systems which include attributional concepts to explain illness causality. Although the ailments are common to all human societies, their types, the ways they are diagnosed and treated depend on how people regard them, and normally varies from one society to another^23^. Consequently, to adequately comprehend the aspect of mental illness, one needs to understand the cosmological assumptions that shape the cultural values perceptions of people, particularly with regard to notions of cause and effect. Therefore, to achieve the aim of the present study of exploring various types of mental illness and their treatment approaches by Swati THs who are of African descent, we followed the Afrocentric theoretical framework. This framework as a philosophy and theoretical paradigm places African ideals, values and philosophies at the centre of an analysis that involves African culture and behaviour^24^.

Treatment methods employed by THs of various cultures in South Africa include: ritualised divination and explanations of aetiology of illness, followed by the following prescribed rites, and herbal medication (muti) which is made from plant, animal and minerals imbued with spiritual significance^25^. Furthermore, purification practices which encompass bathing, vomiting, steaming, nasal ingestion, enemas, and incisions^26^, as well as performing summoning rituals by burning plants like imphepho (Helichrysum petiolare), dancing, chanting, channelling or playing drums are performed^27^. Traditional healers also provide psychosocial intervention which is aimed at relieving distress and improving symptoms in common mental disorders such as depression and anxiety^28^. However, relatively little information is known about the approaches used by Swati THs to treat mental illnesses. Mbwayo et al.^28^ observed that culture is an important factor in healthcare, and that each culture has its own unique conceptualization about illness and treatment approaches. Therefore, the aim of the present study was to explore the different types of mental illness treated by Swati traditional healers, their opinions on the causes of mental illness, and the intervention methods they use to treat patients presenting with mental illness.

## Methodology

### Research design

Phenomenology was adopted as a research design for the present study. In particular, the researchers adopted Martin Heidegger's hermeneutic phenomenological approach which requires the researcher to immerse himself/herself within the phenomenon under investigation and to accept that it is not possible to set aside one's presuppositions and beliefs^29^. Immersing oneself within the phenomenon ensures that the people's lived experiences, events or situations are described in rich detail, taking into account the social, cultural and historical contexts^30^. Hermeneutic phenomenology was therefore considered appropriate for the present study as it allowed the researchers to understand and describe mental illness as perceived by Swati traditional healers themselves.

### Study area and population

**Figure 1:**
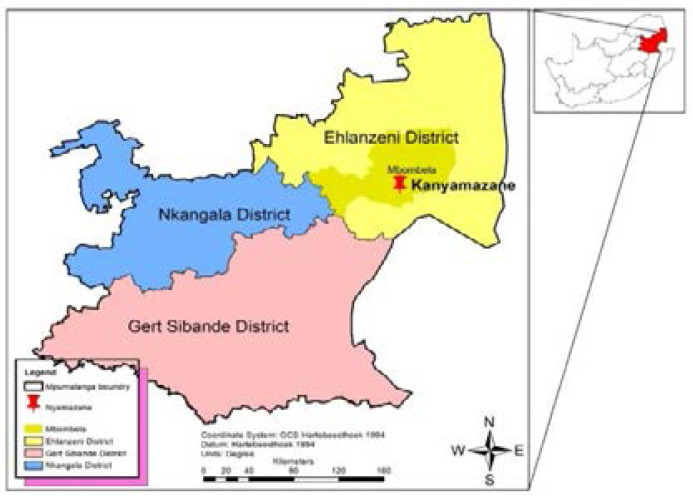
Map of Mpumalanga Province indicating surveyed area

The present study was conducted in Kanyamazane peri-urban township, located in Mpumalanga Province of South Africa (Figure 1). This township is situated within Ehlanzeni district, roughly 30km east of Mbombela (formerly known as Nelspruit city); the capital of Mpumalanga.

The studied area is a cultural home of Swati speaking people who constitute over 98% of the entire population^31^. Swati is a distinct ethnic group closely related to the African Nguni groups, namely Xhosa, Zulu and Ndebele. According to Green^33^, there are two basic types of THs amongst the Swati: the diviner (Sangoma/inyanga) and the herbalist (Lugedla, inyanga yemits).

### Ethical considerations and sampling

Prior to data collection, the researchers obtained permission from the ethics committee of the University of Limpopo (Ethical Approval Number: TREC/FHM/45/2013). Traditional healers who specialise in mental illness treatment were identified using snowball and purposive sampling. In this regard, a traditional healer who specialises in mental illness (and known tothe first author) was approached, and requested to identify other traditional healers specialising in the same field. After the first traditional healer was interviewed, the second traditional healer was approached, based on the recommendation of the first one. The same procedure was followed with all the other traditional healers till a total number of ten was sampled and interviewed. The aim of the study was explained to the THs using iSiswati, the local dialect. The ten THs who agreed to participate in this study were requested to sign a consent form prior to data collection. The researchers respected the privacy, confidentiality, anonymity, cultural sensitivities of the participants who were informed that participation was voluntary.

### Data collection procedures

Data were gathered from the THs via qualitative research techniques (from January 2016 to July 2016), using semi-structured interviews. Overall, the interviews were designed to gather in-depth information relating to social characteristics of THs (i.e. profession, age and number of years working as mental health care practitioners), and the treatment approaches/ methods they use to treat these illnesses. Information relating to the latter encompassed different types of mental illness, THs opinions on the types and causes of mental illness, and the intervention methods they use to treat patients presenting with mental illness.

Each healer was interviewed independently using siSwati language. Credibility was established in the study by reviewing literature on the subject matter. Given that the interviews were recorded in Swati language and later translated to English, two Swati translators double checked the transcripts to avoid omitting any original expressions by the participants.

### Data analysis

Data was analysed using Braun and Clarke's six steps of thematic analysis^33^. The six steps are: familiarising oneself with the data; generating initial codes; searching for themes; reviewing themes; defining and naming themes; and, producing the report. What we have presented in the results section below are the consolidated themes that have emerged as we followed the six steps of data analysis.

## Results

### Social characteristics of participants

A total of ten (10) THs (female = 6; male = 4) belonging to Swati ethnic group participated in this study. Concerning their profession, there were more diviners (= seven, or 70%) than herbalists (= three, or 30%), and both belong to the Traditional African Religion. Female diviners were many (= six or 85.7%). All THs (= three) practicing herbalism were male. Generally, all THs who participated in this study were over 30 years of age, and have been practicing and treating mentally ill patients for more than 10 years. Educationally, most THs completed grade 12 and secondary schooling (Grade 8–11) (= four for each, or 40% for each). The rest of the THs included those who had tertiary qualification and no formal education (= one for each, or 10%).

### Traditional treatment methods of mental illness

A total of seven mental diseases namely adjustment disorders, depression, mental illness due to ancestral calling, mental illness due to bewitchment, mental illness due to breaking of taboos, psychotic disturbance and substance induced mental illness were documented as being treated by Swati THs practicing in the Kanyamazane peri-urban township, in the Mpumalanga Province of South Africa. With the exception of mental disorder due to ancestral calling, which was diagnosed by 70% (n=07) participants, the remainder of the ailments were treated by all questioned THs. According to these healers' patients show signs of improvements within few months of treatment of the aforementioned disorder. Overall, treatment given to the patients is holistic, addressing the body, soul and spirit, and not exclusively focused on a single disorder. It should be stated that Swati THs accommodate mentally ill patients in their homes for treatment until they recover. When these healers were asked about the different treatment procedures employed for the referred disorders, nine themes emerged namely: mental illness due to ancestral calling, psychotic disturbance, substance induced mental illness, depression and adjustment disorders, mental illness due to bewitchment, mental illness due to breaking of cultural taboos and referral. It should be stated that amongst the diverse traditional methods employed by THs for mental illness, some specifically cleansing, herb burning, drumming, and ritual enactment (cleansing rituals, purification practices, offering sacrifices to appease ancestors) were perceived by interviewees as rituals. It is noteworthy that certain mental illnesses are cured through the performance of some of the referred rituals. When asked about the significance of rituals in the treatment of these ailments, participants (100%) replied: “Rituals may positively influence the mental health of an individual”.

Rituals generally encompasses slaughtering of an animal preferably a goat to appease the ancestors for forgiveness. Three (30%) of THs said: “this normally occurs when a patient has not shown good conduct”. Therefore, they have to make peace with their ancestors. In addition to the killing of a goat, some THs (70%) reported burning of herbs as useful in calling the ancestors in order to create harmony and peace in patients' mind by banishment of evil spirits, ultimately ensuring protection of physical and spiritual bodies.

### Approaches used to treat mental illness

It should be emphasised that prior diagnosis of seven mental illnesses recorded in this study, 70% THs use divination and thereafter assess patients (=10). Diviners articulated that prior commencing with treatment, they seek reverence from their ancestors via divining bones while wearing special traditional garments and beads. Generally, ancestors were consulted by interviewees for advice and guidance about the problems of the patient, and specific method of treatment to utilize. The following extracts corroborate THs explanations: “When a person is brought to my compound, initially I throw bones (ngiphengula nge tinhlola) to access the advice of ancestors so that they can lead to appropriate treatment course of action to follow”

After consultation with ancestors, THs also assess a patient' s condition before treating it. One healer said: “I observe the patient's behaviour first; his/her actions will direct me as to how bad the illness is”. He further said: “in some cases, I question family members to obtain a full description of the current concerns and a history of the illness of the patient”. This according to participant, is usually done on patients who cannot articulate their problems. The various themes that emerged from in-depth individual interviews with THs regarding the treatment of mental illness are described below:

### Mental illness due to ancestral calling

This disorder was treated by THs through initiation (kwetfwasa). Initiation was employed by 60% of THs as a healing method for patients presenting with mental illness caused by the ancestral calling to become THs. Initiation is a training program for one to become a traditional healer. According to THs a person receiving this kind of treatment will only recover once he/she has accepted the calling and completed training. The following quotation illustrates this: “calling to become THs is a gift from ancestors and for one to appease them; he/she must undergo training known as kwetfwasa in SiSwati language”. Failure to do so will result in more illnesses, even death, until the person gives in and goes to be trained”. A trainee healer trains formally under another established TH (gobela) to learn all the basics of traditional healing for a period of months or years. The training generally involves learning humility to the ancestors, purification through steaming, washinin the blood of sacrificed animals, and the use of muti.

As part of the initiation process, drumming technique was specified by 70% of THs as imperative way of summoning the ancestors to assist in healing patients diagnosed with mental illness due to ancestral calling. During drumming patients are instructed to dance, and this according to the THs, aid in the reconstruction of the patient's physical, social, and spiritual environments, and to communicate with his/her ancestors. To express this, one of the THs said during an interview: “Sometimes drums are beaten to allow ancestors to take possession and communicate directly with the patient; they provide specific revelation about the problems of the patient”. Another participant added: “I use drumming as therapeutic strategy to gain insight into the patients' illnesses, and ultimately to understand their mental illnesses”. The methods employed when treating mental illness brought obout by ancestors are said to have spiritual significance.

### Psychotic disturbance

Psychotic disturbances such as delusions and hallucinations were also identified as symptoms that accompany mental illness. Techniques used to treat psychotic disturbances included herbal medication and nasal inhalation. It should be highlighted that THs believed that hallucinations may mean visitation by ancestors to provide useful guidance while some believed that hearing voices could be evil intrusions. Therefore, as part of treatment protocol traditional herbal medication and nasal ingestion were used by THs as phytotherapies to assist patients recover. Herbal remedies (timbita), usually include combination of roots obtained from numerous plants, and animal substances. The prepared mixture from these materials is boiled and the resulting steam is inhaled by patients, and cool extracts is used to bath the body. These actions according to THs cleanses out all evil intrusions in patients and ultimately heal their psychotic disturbance. Treatment of this mental disorder through intranasal administration by THs involved the insertion of certain medication in to the patients' nose. According to THs intranasal drug delivery is more effective in the treatment of psychotic disturbances. This is supported by the following quotes: “Medicine given to patients diagnosed with psychotic disturbance via nose are more effective as they go straight to affected central nervous system and repair them instantly”. Generally, the correct utilisation of traditional medicine in healing psychotic disturbance was stressed by THs. The following quotes from one of the THs are relevant: “Patients must be given the correct treatment because wrong prescriptions might lead to other problems such as mortality. That is why we cannot go around telling people to go harvest plants themselves, we have the expertise and ancestors' guidance to exploit the correct species”. There are certain sacred rituals known to us only that must be performed prior harvesting of plant species to ensure that remedies prepared from such species work effectively”.

### Substance induced mental illness

Patients affected by mental illness resulting from alcohol and drug abuse are also kept at the healer's place for rehabilitation. To treat this mental ailment, THs follow multiple therapies comprising of herbal remedies, nasal ingestion (combination of herbs and different animal extracts), rehabilitations and total withdrawal. Nasal ingestion and herbal remedies were employed by all questioned THs, and the latter two techniques were followed as supplementary treatment procedures by 70% of THs. The utilisation of herbal therapies and nasal ingestion as treatment for substance induced mental illness follow a comparable procedure as described under psychotic disturbance. However, the only exception was the specific plants and animal materials used to prepare medication. One Interviewee emphasized this as follows: “Traditional remedies vary according to mental illness to be treated, and we use different floral and faunal parts for dissimilar mental disorders”. Rehabilitations as traditional method of treating mental challenges was exclusively employed for substance induced mental illness. Treatment via rehabilitation as disclosed by THs is primarily meant to restore well-being of an individual diagnosed with this illness. This was evident in the following statement communicated by interviewed THs: “we stay with our patients in our houses to assist them with respect to their mental stability and normality. Furthermore, we provide a supportive environment to ensure full recovery of our patients”. According to the healers, total withdrawal and rehabilitation process are very helpful to patients, especially those who experienced substance induced mental illness. After these process; “herbal remedies are fused in the drug addict's tea and prescribed to the patients. Similarly, drops of some of medicinal preparation is poured into alcohol addict's beer for consumption. Sometimes the herbs are mixed with the dagga, rolled in newspaper and smoked.

### Depression and adjustment disorders

Depressive conditions were treated by all THs who participated in this study. The most common technique of treating this disorder was via herbal prescriptions taken orally as extracts. However, some THs (n=07, 70%) augment this with psychotherapy, wherein healers interrogate patients about their thoughts as well as feelings, and subsequently provide counseling on how to deal with negative thinking or problems and adopt better ways of life. Generally, mentally ill patients diagnosed with severe depression were admitted by THs in their homes until they were totally cured.

Similarly to depressive illness, adjustment disorders were also diagnosed by all probed Swati THs who use counselling and herbal remedies as treatments. However, the latter was a principal method of healing adjustment disorders valued by all THs. It should be stated that herbal medication for this disorder was exclusively prepared by burning the herbs. According to THs, smoke-based remedies are most effective in healing the referred disorder because they are delivered to the brain rapidly, relieving all stress-related conditions. In addition to the utilisation of smoke-based remedies, some THs also counsel patients as part of the treatment procedure.

### Mental illness due to bewitchment

This disorder was treated by all THs in the present study using cleansing and incision (kugata) techniques. Traditional healers perceive mental illness due to bewitchment to be caused by witches who use evil mystical power with the intention to kill someone or distort their behaviors.

Treatment of mental complaints caused by d bewitchment using the above-mentioned techniques in this study were wholly traditional. Participants said: “a patient's body is incised to allow the causative forces to leave the body”. One participant added: “The dirty blood must come out of patients' body, to complete the healing process, which is why sometimes I incise my patients”. Likewise, steaming (kufutsa) (made from herbs and animal materials) is also believed to cleanse the body and eliminate evil intrusions. This is supported by the following quotes stated by THs: “patients are ordered to take off most of their clothes and steamed under a blanket. The steaming, opens up all body pores and allow medicine to infiltrate patients' body, thus chasing away all evil forces”.

Furthermore, as part of treatment of mental illness due to bewitchment, interviewees also employ the technique of being mediators to negotiate for the health of their patients. According to THs (n=8, 80%), sometimes people transgress others by stealing from them who in turn revenge by bewitching them to become mentally ill. In this regard, THs assist such patients by acting as mediators between the victims and the perpetrators to plead with the victims to break the curses casted upon the perpetrator. Perpetrators are urged to compensate victims as a way of showing remorse and asking for forgiveness. Traditional rituals are performed to break the curses to cure the patient's mental illness. The following statement by 60% (n=6) of THs support this: “A person who has stolen property from another person and was bewitched as a result must be reconciled with the victim. I accompany the perpetrator to the victim's family to negotiate forgiveness, we perform some sort of reversal rituals”.

### Mental illness due to breaking of cultural taboos

All participants in this study treated mental illnesses caused by transgressing cultural taboos. Treatment procedures employed by participants generally centered round ritual enactments, and comprises cleansing rituals, offering sacrifices to appease ancestors and purification practices. The following statements are relevant: “we slaughter a goat as an offering to ancestors so that they forgive our patients for breaking cultural taboos”. Cleansing rituals encompass washing, steaming, induced vomiting, inhaling herbs, offering sacrifices to gods to purify patients from their cultural misconducts.

Generally, ritual enactments especially offering of sacrifices to the ancestors were mentioned by THs as significant folk customs that ensure complete healing of mental ailment due to breaking of cultural taboos.

### Referral

Although THs emphasised that there are aliments that can be cured solely through their intervention, they also acknowledged Western trained health care providers, hence they sometimes refer their clients to seek help from them. This is corroborated by one of the THs statement: “Since I do not have the screening tools for some illnesses, for example, I cannot check the severity of a patient's high blood pressure, I refer my patients to the Western doctors for medical examination. They will come back to me with the feedback. I will then commence treatment”.

## Discussion

The burden of mental sickness coupled with a dire shortage of Western-trained psychiatrists and government owned mental facilities is prevalent in South Africa, and this is depicted by the recent “Life Esidimeni” saga, which resulted in more than 140 patients dying after the government terminated an outsourced care contract with specialized private psychiatric institution (due to high financial cost), and discharged patients to places that could not give them the care they required. This heart-breaking saga, which left the country in shock, indicate quite clearly the need for more and affordable mental health service providers. Interestingly, the role of THs as complementary health service providers specifically as inexpensive and easily accessible mental health workforce in rural areas of South Africa^34^, ^35^ and other African countries^26^, ^27^, ^36^, ^37^ cannot be overemphasized. The present study offers insight into the role played by Swati THs in the treatment and management of mental disorders in the Kanyamazane peri-urban township (Mpumalanga Province, South Africa).

Ten THs providing mental health care services, comprising mainly of female compared to male were questioned. Most of these THs were diviners (70%) with herbalists constituting just 30%. Similar finding was reported amongst THs of other ethnic groups in South Africa^38^,^39^. The supremacy of diviners in the treatment of mental illnesses in our study might be ascribed to the various divination treatment approaches used, which are primarily ancestral gift for diviners than herbalists^40^. All interviewed THs were over 30 years old and have been treating patients diagnosed with mental illness for more than 10 years. This finding suggest that those THs possess a considerable wealth of experience with regard to the various aspects of mental health care. Dalal^41^ found that older THs with more working experience have accumulated great healing knowledge and skills during years in practice. Thus, it can be said that questioned Swati THs are more likely to deliver high-quality mental health care as they have acquired sufficient experience and techniques, which allows them to not only rely on ancestral spirits guidance for treatment of mental disorders but also to use more experience-based knowledge to make decision with respect to their patients' mental health care needs. Similarly, an overwhelming majority (90%, n=9) of THs that were interviewed had a certain level of education, which allows them to write accurate prescriptions and dosage strength of certain treatments (i.e. herbal medicine) to family members caring for psychological patients.

Different types of mental illness ranging from common ones such as adjustment disorders, depression, and substance induced mental illness, to cultural causes of mental illness including psychological illness due to ancestral calling, bewitchment, breaking of taboos, and psychotic disturbance were treated by Swati THs. This finding was expected due to the fact that all THs belong to the Traditional African Religion, which encompasses belief in a supreme creator, spirits, veneration of the deceased, use of magic and traditional African medicine^42^. Nevertheless, the diversity of diseases treated shows that interviewees play an important role in mental health care in the studied area. It is worth stating that some of the referred psychological complaints diagnosed by Swati THs are similar to those commonly treated by THs of other cultures in South Africa and other African countries. For instance, our findings are in line with Crawford and Lipsege^43^ who reported the treatment of psychological ailments due to sorcery by Zulu THs. Audet et al^44^ also found that Tsonga THs practicing Bushbuckridge area of Mpumalanga province in South Africa, also treat substance induced mental illness. Similarly, psychotic disturbance is also treated by THs in Egypt^45^. Traditional healers in Kenya also treat depression^46^. Similar finding was reported by Abbo^47^ amongst THs providing mental health services in two districts of Eastern Uganda. The treatment of the cultural causes of mental illness in the current study was expected due to the fact that patients and their families view its origins to have cultural root causes and not biological or genetic causes as allopathic practitioners may assert^48^. For instance, in Ghana most people prefer THs services for the treatment of mental illnesses because their approaches are consistent with hegemonic cultural explanatory models of mental disease aetiology^49^. Generally, it should be stated that all questioned THs not only specialise in the treatment mental disorders but also treat a wide range of other conditions. Importantly, as part of the treatment of the above-stated mental conditions, Swati THs also treat other affliction not diagnosed as mental illnesses. They believe that compete healing of patients in their culture should address any ailment including those that are not related to mental illnesses. Hence, they supplement divination (i.e. throwing of bones), with history-taking and physically observation of patients to ensure treatment given is holistic and address the body, soul and spirit, and not only a single mental sickness.

Findings of the present study further revealed that Swati THs use different treatment modalities namely herbal remedies, nasal ingestion, rehabilitations and total withdrawal for the earlier alluded mental illness. These are the common traditional treatments approaches to mental illness employed by THs of other cultures in South Africa^50^, ^51^, ^52^, ^53^, ^54^ and elsewhere^55^. However, the use of some of previously mentioned modalities by Swati THs primarily depended on the type of disease and the cause, but THs mainly integrate them for treatment of a single mental disorder. It is worth echoing that prior to commencement with any mental disorder treatment, most THs seek reverence from their ancestors (i.e. for advice and guidance about the problems of the patient, and specific method of treatment to utilize) via divining bones while wearing special traditional healing regalia. As highlighted earlier, this is followed by history-taking and physical observation of patients. The role of ancestors in the traditional healing of psychological disorders by African THs belonging to diverse cultures is constantly emphasised in literatures^52^,^53^. Therefore, the involvement of ancestors as first action of treatment of these disorders in this study was expected.

While the treatment approaches of most mental disorders by Swati THs are culturally accepted and cannot be explained by allopathic approaches, some make sense from scientific perspective. For instance, herbal remedies were used as front-line treatment for depression and adjustment disorders supplemented by counselling. These practices are comparable to those followed in the scientific medical fraternity. Although, one can argue from a cultural stand point that the use of herbal medication by Swati THs as antidepressants dates back, and was tested over time and proven to be effective, there is a need to evaluate their efficacy and toxicity. The positive outcomes of this explorations can be used as bridge towards the integration of keeping patients affected with mental illness resulting from alcohol and drug abuse at the healer's place as recorded in this study for integrated treatment (i.e. herbal remedies, rehabilitations and total withdrawal) is also proven systematically to be effective in patients suffering from mental problems caused by drug abuse^56^. As part of treatment of this patients Swati THs prescribe some herbal preparations and administered them to patients as anti-drugs medicines. Similar finding was reported by Mashamaite^52^ who explored treatment of mental illness by Bapedi healers in Moletjie area located in the Capricorn district, Limpopo Province (South Africa). The purpose of rehabilitating patients diagnosed with mental diseases resulting drug abuse in the current study was meant to restore well-being of patients. In this regards, THs provide patients with skills for reducing or abstaining from substance use. The logic for the use of integrated treatment is that it simultaneously addresses more than one interwoven disorders, and this approaches has been found to be effective in treating psychological problems emanating from substance abuse^57^,^58^. Therefore, the approach of treating this problem by THs in this study share some common principles with Western approaches. Similarly, the use of drumming (ritual drum ceremony) by Swati THs to treat mental illnesses due to ancestral calling which is also a common practice amongst THs of other culture in South Africa^59^,^60^, is partly supported by a scientific study which found that 39 mental health patients demonstrated recovery following engagement with a programme of group djembe drumming in the United Kingdom^61^. Similar findings were reported by Fancourt et al.^62^. Therefore, the utilisation of drumming by Swati THs in the treatment of mental illness due to ancestral calling have psychological benefits.

It is interesting to note that THs in this study refer their mentally ill patients to local Western trained health providers to seek help (which they cannot provide such as checking high blood pressure, amongst other) from them. This finding is similar to that reported by other researchers in South Africa^52^,^53^,^63^. On the contrary, AE-Ngibise et al.^49^ found that THs in Ghana who treat mental illnesses appeared to be reluctant to engage with ‘conventional’ medical practitioners due to the solidarity and camaraderie they felt with other healers. Subsequently, they expressed a preference for referring a client to another healer rather than to a Western doctor. The external referral systems of mental patients to Westernised health-care providers as observed in the present study should be viewed as a step forward towards possible professional collaboration between local Swati THs and allopathic health practitioners in the treatment of psychological disorders, although this might take years to achieve due the complexity of the two health care systems. However, to speedup this process, both healthcare providers must actively reciprocate, this ultimately will make them fill appreciated and recognised.

Testimonial-based evidences also exist indicating the effectiveness of some of the treatment protocol followed by Swati THs to treat mental illness due to bewitchment^64^ and breaking of cultural taboos^65^,^66^. These studies reported the recovery of patients who were treated with some methods followed by interviewees in the current study, thus supporting the therapeutic potentials of such techniques for mental health. Despite this evidence, policy makers and health professionals remain skeptical about the acceptance and integration of the referred techniques,uestioning the authenticity of some of their elements such as the use of ancestral spirits and sacrifices which could not be subjected to rigorous empirical methods^48^,^67^.

Based on the results of the present study, it is therefore important to understand the practices of THs from their own cultural context. This is consistent with the Afrocentric theoretical framework that the researchers adopted for the present study. Based on this assertion, it is suggested that any attempt to examine the methods employed by THs, including efforts to work collaboratively with them, should take into account the cultural ideologies that inform their practices.

## Conclusion

The present study has shown that Swati THs may play an important role in addressing the mental health care needs in the studied area by offering both culturally and scientifically appropriate treatments and would therefore, help complement the services provided by health care professionals. The latter is primarily based on the fact that THs admit patients in their own homes until they recover. Generally, the unique and effective positive aspects of traditional healing approaches documented in this study should be recognised and incorporated into the local mainstream of the primary health care system.

Nevertheless, findings from this study should be interpreted with the following limitations. Firstly, translating the interview data from Swati to English dialect might have led to omissions or inappropriate substitutions of the original rich material provided by the THs. Secondly, the survey relied only on THs' subjective accounts of the treatment of mental illness. Other people (for example, patients) were not interviewed. Consequently, it is acknowledged that the present study provided a one-sided interpretation of the treatment of mental illness. Thirdly, the study was conducted on a limited sample of Swati THs practising only in one peri-urban township in Mpumalanga of South Africa. Lastly, the authors have, in a number of instances, used Western terminology such as depression and adjustment disorders. This approach was necessary in order to make the language accessible to Western scholars.

A comprehensive survey involving larger sample of THs is recommended so as to fully understand the role of Swati THs in the treatment of mental illnesses. Similarly, a broad enquiry involving patient's subjective experiences in utilising Swati traditional mental health treatment and its efficacy should be a subject for future study. Such future studis could provide greater insight into the various approaches used by THs for mental diseases, and perhaps, open doors for possible closer collaboration between THs and western trained mental health practitioners.
